# Multifunctional Graphite Nanosheet–Hydrophilic Epoxy Anticorrosion Coatings via Size Confinement of Exfoliated Graphite

**DOI:** 10.3390/polym17131803

**Published:** 2025-06-28

**Authors:** Huachao Ma, Xuyang Zhang, Dongxing Zhang, Yizhan Peng, Detian Wan, Tai Peng, Kuilin Lv

**Affiliations:** 1 College of Materials Science and Engineering, Jiamusi University, Jiamusi 154007, China; m18769082751@163.com (H.M.); 18753019632@163.com (Y.P.); 2 China Testing & Certification International Group Co., Ltd., Room, Beijing 100024, China; 18736768339@163.com (X.Z.); zhangdongxing@ctc.com (D.Z.); dtwan@ctc.ac.cn (D.W.); 3 School of Civil and Transportation Engineering, Beijing University of Civil Engineering and Architecture, Beijing 102616, China

**Keywords:** mullite/kaolin coatings, size effect, anticorrosive coatings, mechanical wear resistance, graphite nanosheets

## Abstract

To assess how the graphite nanosheet size affects the performance of hydrophilic coatings, graphite nanosheets of various sizes were added to a mullite/kaolin epoxy (EP) coating. The experimental results indicated that the mullite/kaolin EP coating enriched with graphite nanosheets (1.01 ± 0.1 μm) exhibited the highest impedance value of 9.18 × 10^7^ Ω cm^2^, demonstrating the best performance after 2880 h of exposure to salt spray. This implies exceptional wear resistance. Appropriately sized graphite nanosheets can create excellent nanonetworks that cover micropores, which cannot prevent the diffusion of corrosive media and provide excellent mechanical properties to coatings. The results of this study serve as a reference for the industrial application of graphite anticorrosive coatings.

## 1. Introduction

Corrosion is the degradation of a material owing to chemical reactions with the environment [1,2,3]. In metal pipes and equipment, corrosion gradually deteriorates structures, leading to material discharge, leaks, and environmental contamination from raw materials and products [4]. Managing and mitigating the effects of metal corrosion are generally more feasible and practical routes than attempting completing eradication [5,6].

Epoxy (EP) resins are commonly used in anticorrosion coatings owing to their excellent barrier properties. However, after curing, microcracks can develop in the EP coating, allowing corrosive ions to penetrate the epoxy layer [7,8,9]. Barrier substances are generally added to the EP coating to fill these microcracks, thereby improving the corrosion resistance, elastic stability, and overall durability of the coating [10,11,12]. Mullite is widely used in environmental barrier coatings and refractory materials owing to its outstanding properties, including high-temperature stability, corrosion resistance, low thermal expansion coefficient, and excellent thermal shock resistance [13,14,15]. Although it is primarily used in the sintering of ceramics and ceramic coatings, its application in anticorrosion coatings has been underexplored. Therefore, we chose to use mullite as the inorganic substance in the coating to investigate its anticorrosion properties when incorporated into the formulation [16,17,18]. Kaolin has small particles and a large surface area, which can improve the interface bonding strength between epoxy resin and the substrate through the physical anchoring effect, and it is a commonly used corrosion resistant coating filler. Mullite as a ceramic powder itself has excellent acid and alkali corrosion resistance, its crystal structure is dense, its surface activity is low, and it can effectively block the penetration of corrosive media, but it is mostly used in ceramics, and it is rarely used in anticorrosion coatings. The composite use of mullite and kaolin can form a complementary effect and improve the corrosion resistance of the coating itself. If mullite and kaolin are not added, the salt spray resistance time of the simple epoxy resin coating can only reach 48 h, and different coatings will have a rapid corrosion effect in a short time, which cannot reflect the best corrosion resistance of the coating. Therefore, mullite and kaolin are considered as fillers for epoxy coatings.

Graphite possesses high electron mobility, optical transparency, and excellent thermal conductivity, rendering it a popular additive in hydrophobic coatings [19,20,21]. Yang Z et al. [22] prepared a superhydrophobic coating with self-cleaning ability, mechanical wear resistance, and chemical stability by exfoliating fluorographene nanosheets in a liquid phase. Additionally, Lv K et al. [23] demonstrated the influence of particle size on corrosion resistance by creating graphite nanosheets of varying sizes and incorporating them into hydrophobic coatings. Ma H et al. [24] prepared a new type of superhydrophobic anticorrosion coating by simplifying the incorporation of graphite nanosheets and verified the application of the size effect in hydrophobic coatings. Although the hydrophobic properties of these coatings can offer high corrosion resistance, verifying the size effect in hydrophobic coatings may introduce large errors [25,26,27]. To mitigate this issue, we used a hydrophilic coating to assess the size effect of graphite nanosheets on corrosion resistance.

For this purpose, we prepared solutions containing varying sizes of graphite nanosheets via ball milling and gradient centrifugation. New hydrophilic coatings were synthesized using mullite, kaolin, and EP resin, and different hydrophilic corrosion-resistant coatings were prepared by incorporating solutions containing various graphite nanosheet sizes to the new hydrophilic coatings. Specifically, microkaolin, mullite, and graphite nanosheets of varying sizes were integrated into the WEP coating (designated as EG_x_/A_4_C_6_EP, where X indicates the centrifugal rate, A represents mullite, and C stands for kaolin). Through salt spray and electrochemical tests, the effects of graphite nanosheet size on the corrosion resistance of four hydrophilic coatings were assessed and verified.

## 2. Materials and Methods

### 2.1. Sample Synthesis

Graphite nanosheets of different dimensions were synthesized via wet ball milling, followed by gradient centrifugation [23]. A mixture of EG (graphite powder) (4 g), 2 and 0.2 mm zirconium dioxide microspheres (mass ratio 1:1), and NVP (N-vinylpyrrolidone) (100 mL) was added to a 250 mL ball mill tank and subjected to continuous ball milling for 175 h. After milling, the dispersed material was collected, and the solution was divided into four parts. One-quarter of the solution was centrifuged at 350 r min^−1^ for 10 min, and the supernatant was collected and stored as sample EG_350_. The remaining three samples were similarly centrifuged for 10 min at 800, 1500, and 3000 r min^−1^, respectively. The supernatant was collected and stored as the EG_800_, EG_1500_, and EG_3000_ samples with concentrations of approximately ≤0.065 wt%.

A mullite/kaolin slurry with a 4:6 (mass ratio) ratio was evenly mixed using wet ball milling. Castor oil was added as a dispersant, followed by the addition of graphite nanosheet dispersions of different sizes (5 wt%). The coating emulsion was then obtained through continuous ball milling for 4 h.

In previous studies, we examined the effect of different graphene nanosheet concentrations on the corrosion resistance of coatings. Our findings indicated that optimal corrosion resistance was achieved at a graphene nanosheet concentration of 0.075 wt% [23]. Therefore, in this study, we directly incorporated 0.075 wt% graphene nanosheets for experimentation. To produce the hydrophilic coating, a 3.3 g mixed solution was extracted, and EP (epoxy resin) and PA (polyamide curing agent) were added in a 1:1 mass ratio. The pretreated Q235 steel plate was then uniformly coated using a 150 µm wire rod and cured at room temperature for 24 h, resulting in the EG_x_/A_4_C_6_EP coating. The thickness of the prepared coating is 60 ± 10 μm.

### 2.2. Characterization

Field emission scanning electron microscopy (SEM, Model S-4800, Tokyo, Japan) and high-resolution transmission electron microscopy (HRTEM, JEOL, Model JEM-2100F) were used for material characterization. SEM analysis was performed at a high voltage of 20 kV, with a focal point (spot size) of 5.5, a magnification of 1200×, and a working distance of 13.3 mm. HRTEM was performed at an acceleration voltage of 100 kV, a magnification of 5000×, and a resolution of 0.1 nm. X-ray diffraction (XRD) analysis was performed using the Terra system (Innov-X, Woburn, MA, USA) with a scanning angle range of 10–80°, a scanning rate of 5°/min, and a copper target. Fourier transform infrared (FTIR) spectroscopy was performed using a Shimadzu IR-21 spectrometer to analyze the sample composition. FTIR spectroscopy was performed with a scanning range of 4000–400 cm^−1^, a resolution of 4 cm^−1^, and 32 scans. Surface properties were analyzed using X-ray photoelectron spectroscopy (XPS) with a Thermo ESCALAB250 instrument, using a Mg Kα X-ray source (1253.6 eV). The measured sample depth and escape depth ranged from 4 to 10 nm. Static contact angle measurements were performed using the OCAH200 instrument (DataPhysics, Feldstadt, Germany).

### 2.3. Electrochemical Measurement

Corrosion resistance was evaluated through electrochemical measurements and salt spray tests. Electrochemical measurements were performed using a DH7000 electrochemical workstation, with electrochemical impedance spectroscopy (EIS) and Tafel polarization tests performed in a three-electrode cell containing 2 M NaCl as the electrolyte (see Supporting Information for detailed procedures). To ensure a comprehensive assessment, salt spray testing was performed to examine long-term corrosion behavior under simulated environmental conditions. Four different coatings were applied to 1 cm × 1 cm Q235 steel plates and immersed in a NaCl solution for 30 days, with observed changes in the coatings used to assess their durability.

### 2.4. Wear Resistance Measurement

The mechanical abrasion resistance of the coating was evaluated using a combination of sandpaper friction tests and scratch resistance tests [28,29,30]. Friction resistance was determined based on the grit size of the sandpaper and the applied load. In this experiment, a 500 g weight was applied, and 150 g hawkish sandpaper was used to define the contact coating roughness. The film was placed in contact with the sandpaper, and together with the substrate, it was subjected to a 500 g load. An external force moved it horizontally along the balance by 3 cm per cycle. Wear measurements were taken every 5 cycles for the first 50 cycles and then every 10 cycles thereafter. After 100 cycles, the surface condition and contact angle of the hydrophilic film were evaluated.

## 3. Results and Discussion

### 3.1. Structure and Morphology

#### 3.1.1. Morphology Characterization of Samples and Coatings

**Figure 1:**
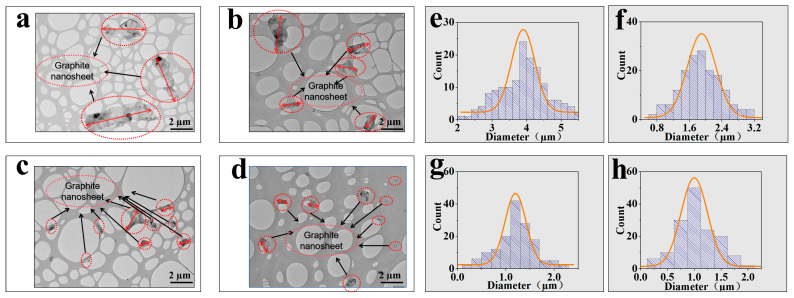
HRTEM images of EG_350_ (**a**), EG_800_ (**b**), EG_1500_ (**c**), and EG_3000_ (**d**). Size statistics of EG_350_ (**e**), EG_800_ (**f**), EG_1500_ (**g**), and EG_3000_ (**h**).

EG_x_ sheet materials of varying sizes were systematically prepared via gradient centrifugation. The TEM results of some graphite nanosheets are presented (Figure 1a–d), and the sizes of the EG_350_, EG_800_, EG_1500_, and EG_3000_ sheets gradually decrease, confirming that ball milling is an effective method for reducing layered materials to different sizes [24]. The morphology of the exfoliated EG_x_ sheets exhibits no signs of fracture or agglomeration, maintaining a relatively uniform size in the transverse dimension. Specifically, measurements on 200 sheets of each type show average diameters and lengths of 4.01 ± 0.15 (EG_350_), 2.01 ± 0.18 (EG_800_), 1.32 ± 0.23 (EG_1500_), and 1.01 ± 0.21 μm (EG_3000_), demonstrating the successful exfoliation of bulk graphite powder into multilayered fragments.

**Figure 2:**
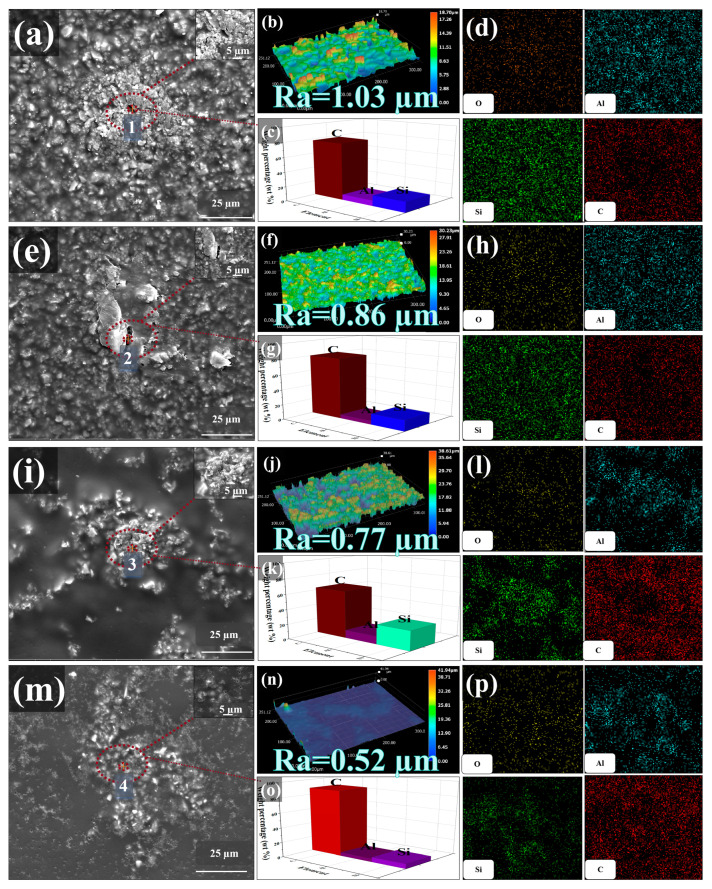
SEM images of EG_350_A_4_C_6_EP (**a**), EG_800_A_4_C_6_EP (**e**), EG_1500_A_4_C_6_EP (**i**), and EG_3000_A_4_C_6_EP (**m**). Roughness test results of EG_350_A_4_C_6_EP (**b**), EG_800_A_4_C_6_EP (**f**), EG_1500_A_4_C_6_EP (**j**), and EG_3000_A_4_C_6_EP (**n**). Elemental content images of EG_350_A_4_C_6_EP (**c**), EG_800_A_4_C_6_EP (**g**), EG_1500_A_4_C_6_EP (**k**), and EG_3000_A_4_C_6_EP (**o**). EDS mapping images of EG_350_A_4_C_6_EP (**d**), EG_800_A_4_C_6_EP (**h**), EG_1500_A_4_C_6_EP (**l**), and EG_3000_A_4_C_6_EP (**p**).

To examine the effect of the graphite nanosheet size on the hydrophilic coating, a new EP resin coating with a hydrophobic angle of 59.92° was prepared using mullite and kaolin as fillers. The SEM results, shown in Figure S1, reveal that the EP coating contains numerous dense micropores, which can facilitate the penetration of corrosive particles, leading to their easy contact with the substrate and corrosion reactions. In the process of ball milling, large graphite powder fragments are exfoliated into nanosheets of varying sizes owing to shear and compression forces exerted by large and small ZrO_2_ balls. Graphene nanosheets of varying sizes were added to the EP coating. As the size decreased, the number of filled pores increased, reducing the roughness of the coating (Figure 2b,f,g,n). Consequently, the contact angle of the coating increases (Figure S2). With the addition of graphite nanosheets, pores in the EP coating gradually decrease (Figure 2a,e,i,m), eventually leading to near-complete pore closure in the EP coating containing EG_3000_ (Figure 2m). A part of the selected coating was subjected to SEM, and the images discussed were not representative. At this point, the hydrophobic angle reaches 68.98° (Figure S2). EDS point scanning (Figure 2c,g,k,o) and EDS mapping (Figure 2d,h,l,p) reveal that C is the most dominant element in the coating’s micropores, while Al, Si, and O are primarily distributed around micropores. This indicates that micropores are the inherent defects of the EP coating. Large graphite nanosheets tend to deposit in these micropores, but they do not completely block these micropores. However, in the EP coating with EG_3000_, micropores are almost completely closed, reducing the porosity of the coating. As a result, O_2_ and H_2_O cannot diffuse through these microchannels to the substrate, thereby enhancing the corrosion resistance of the coating.

#### 3.1.2. Structural Characterization of Samples and Coatings

**Figure 3:**
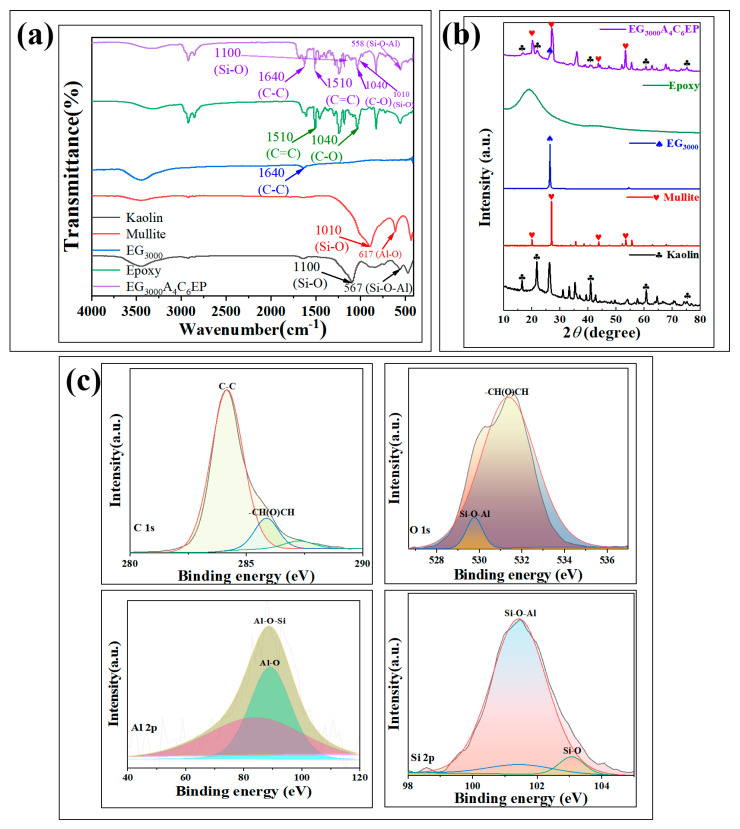
FTIR image of EG_3000_A_4_C_6_EP and samples (**a**), XRD image of EG_3000_A_4_C_6_EP and samples (**b**), and XPS image of EG_3000_A_4_C_6_EP (**c**).

To verify the bonding mechanisms of each component in the coatings, various tests were performed to analyze the coatings and their individual components; the results are presented in Figure 3.

In the infrared analysis of the coating (Figure 3a), the C–C bond vibration peak of the coating at 1640 cm^−1^ matches that of graphite nanosheets. The characteristic vibration peaks of the C=C bond at 1510 cm^−1^ and C–O bond at 1040 cm^−1^ correspond to those of the pure EP resin coating, confirming that graphite nanosheets are physically incorporated into the EP resin coating without forming any new defects. Additionally, the Si–O bond vibration peak at 1010 cm^−1^ in the coating is consistent with that of mullite powder. The characteristic vibration peak of the Si–O–Al bond at 558 cm^−1^ shifts by 9 cm^−1^ to the right compared with that of kaolin, indicating that mullite and kaolin powders serve as matrix fillers without creating new defects. To confirm that each component is completely incorporated into the coating, XRD tests were performed on the coating and its individual components, with the results shown in Figure 2b. In the XRD analysis, the characteristic peaks of mullite were significantly broadened, while those of kaolin were significantly decreased. This transformation occurs because mullite and kaolin are ores mainly composed of Al_2_O_3_ and SiO_2_. Mixing the two powders together as the substrate of the coating filler led to the observed deviation and the weakening of the peaks. The characteristic peak observed by EP was the halo of epoxy resin. The characteristic peaks of graphite nanosheets is clearly present in the coating, with no shift in their peak positions. This further confirms that graphite nanosheets are physically incorporated into the defects of the EP coating through a filling process.

XPS tests were performed on the EG_3000_A_4_C_6_EP coating to validate the FTIR and XRD results, as shown in Figure 3c, with the full spectrum presented in Figure S3. The characteristic peak of the Al–O bond at 90.4 eV and the Si–O bond peak at 103 eV in the coating match the XPS results for kaolin (Figure S4). Additionally, the Al–O–Si bond characteristic peak at 89.4 eV and the Si–O–Al bond peak at 101.5 eV in the coating correspond to the XPS results for mullite (Figure S5). These findings confirm that the mixture of mullite and kaolin functions as the filling matrix. This explains the shift observed in the infrared and XRD data and confirms that no new defects are formed. The C–C bond characteristic peak at 284.0 eV in the coating corresponds with the XRD results for graphite nanosheets (Figure S6). The C 1s characteristic peak of the –CH(O)CH bond at 286.1 eV aligns with the O 1s characteristic peak of the –CH(O)CH bond at 532.2 eV, as well as the XPS results for the EP resin coating (Figure S7). This demonstrates that the graphite nanosheets physically fill defects in the EP coating, without generating any new defects or reactions.

### 3.2. Corrosion Resistance Test

#### 3.2.1. Electrochemical Measurement Result

**Figure 4:**
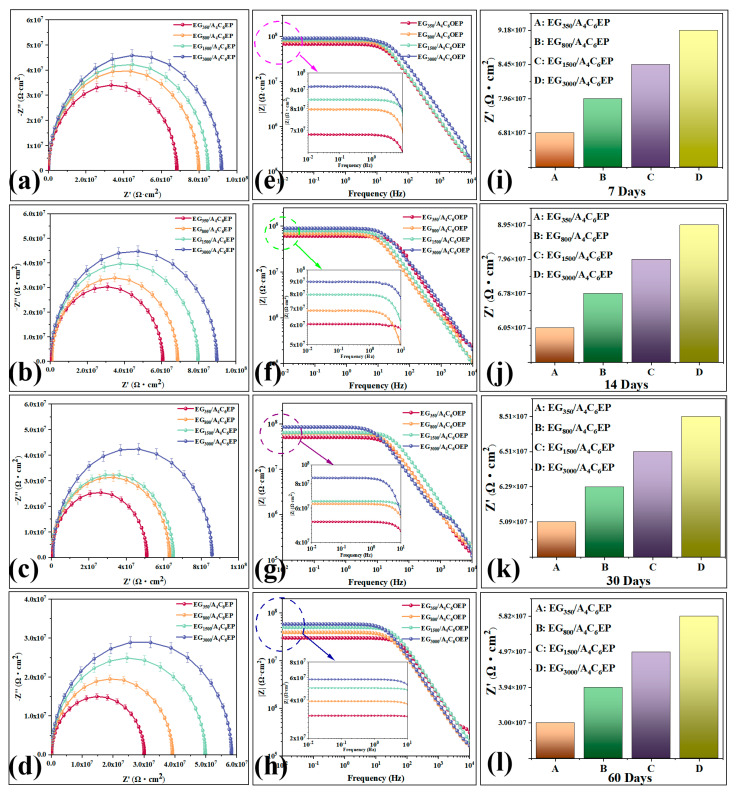
EIS images of EG_350-3000_A_4_C_6_EP coatings at 7/14/30/60 days of immersion (**a**–**d**). Bode images of EG_350-3000_A_4_C_6_EP coatings at 7/14/30/60 days of immersion (**e**–**h**). Impedance images of EG_350-3000_A_4_C_6_EP coatings at 7/14/30/60 days of immersion (**i**–**l**).

To assess the corrosion resistance of the coatings, EIS and Tafel polarization techniques were used to evaluate their protective performance. Additionally, salt spray tests were performed to visually monitor surface changes across different coatings, thereby providing further insights into their corrosion resistance [31]. The coatings were immersed in a 3.5% NaCl solution for various durations, and the resulting EIS and Bode plots are presented in Figure 4. An EIS equivalent circuit diagram is shown in Figure S8, and the detailed results are summarized in Table S1.

In the impedance diagram, it is clear that the system is controlled by substantial charge transfer, and coating resistance closely approximates ideal resistance. As a result, the equivalent circuit diagram R(C(R)) is used (Figure S8), where Rs represents solution resistance, C denotes the charge transfer number, and Rp indicates coating resistance. The magnitude of Rp reflects the impedance diameter of the impedance arc and corrosion resistance of the coating.

In the EIS analysis of different coatings, a larger impedance diameter, for the same immersion duration, indicates the better corrosion resistance of the coating [29]. After 7 days of immersion in the NaCl solution, the impedance semicircle diameters for all four coatings are relatively large (Figure 4a), with the EG_3000_/A_4_C_6_EP coating showing the largest impedance diameter corresponding to an impedance of 9.18 × 10^7^ Ω·cm^2^ (Figure 4i). This represents an almost 50% increase in the impedance diameter compared to that of the EG_350_/A_4_C_6_EP coating, demonstrating that the EG_3000_/A_4_C_6_EP coating has the highest corrosion resistance. After 14 days of immersion in the NaCl solution, the impedance values for all four coatings decrease (Figure 4b). However, the semicircle diameter of the EG_3000_/A_4_C_6_EP coating remains the largest, with an impedance value of 8.95 × 10^7^ Ω·cm^2^ (Figure 4j). Despite the decrease in the impedance value, the order of magnitude remains the same, indicating that the corrosion resistance of the EG_3000_/A_4_C_6_EP coating remains relatively stable and the highest. After 30 days of immersion in the NaCl solution, the impedance diameters of the EG_800_/A_4_C_6_EP and EG_1500_/A_4_C_6_EP coatings become similar (Figure 4c), with impedance values of 6.29 × 10^7^ and 6.51 × 10^7^ Ω·cm^2^, respectively. Meanwhile, the impedance value of the EG_3000_/A_4_C_6_EP coating remains the highest at 8.51 × 10^7^ Ω·cm^2^ (Figure 4k). Only a slight decrease in its impedance value further confirms that the corrosion resistance of the EG_3000_/A_4_C_6_EP coating remains stable, making it the most corrosion-resistant among the coatings. After 60 days of immersion, the impedance diameters of all coatings considerably decrease (Figure 4d); however, the impedance diameter of the EG_3000_/A_4_C_6_EP coating does not decrease by an order of magnitude. This indicates that, even after 60 days in the NaCl solution, the EG_3000_/A_4_C_6_EP coating continues to maintain stable corrosion resistance. Among the four coatings, the impedance diameter of the EG_3000_/A_4_C_6_EP coating remains the largest (Figure 4l), indicating its superior corrosion resistance, which aligns with the trends observed after 7, 14, and 30 days of immersion.

To validate the EIS principles, the Bode test was used to assess the corrosion resistance of various coatings. At a frequency of 0.01 Hz, a higher Bode modulus value indicates greater corrosion resistance [24]. After 7 days of immersion in the NaCl solution, the EG_3000_/A_4_C_6_EP coating exhibits the highest modulus value, reaching 9.2 × 10^7^ Ω·cm^2^ (Figure 4e), considerably exceeding the Bode modulus values of the other coatings and thereby confirming its superior corrosion resistance. At immersion durations of 14 and 30 days, the modulus values of all coatings in the low-frequency region show minimal changes. This indicates that the corrosion resistance of the four coatings remains relatively stable over 30 days. Among them, the EG_3000_/A_4_C_6_EP coating still exhibits the highest modulus value in the low-frequency region (Figure 4f,g), demonstrating its superior corrosion protection performance. After 60 days of immersion, the modulus values of all four coatings in the low-frequency region decrease, indicating a reduction in corrosion resistance. The modulus value of the EG_3000_/A_4_C_6_EP coating in this region decreases to approximately 6.0 × 10^7^ Ω·cm^2^, but it still maintains a relatively high modulus, highlighting its high corrosion resistance and stability. These experimental results are consistent with the findings from Nyquist analysis.

The blank group was immersed in salt spray for seven days, and electrochemical and Tafel tests were conducted. The EIS, Bode, and Tafel graphs of the tests are shown in Figure S9, and the specific values are shown in Tables S1 and S2. The results show that after soaking in NaCl solution for 7 days, the impedance diameter of the A_4_C_6_EP coating is 2.12 × 10^7^ Ω·cm^2^ smaller than that of the EG_350_/A_4_C_6_EP coating, but its own impedance value reaches 4.69 × 10^7^ Ω·cm^2^, proving its own excellent corrosion resistance. And the addition of graphite nanosheets further enhances the corrosion resistance of the coating. In addition, after the EG_350_/A_4_C_6_EP coating was immersed in the NaCl solution for 7 days, compared with the A_4_C_6_EP coating, the Bode modulus value was larger, and Ecoor and Icoor were smaller. This proved that the corrosion resistance of the EG_350_/A_4_C_6_EP coating was greater than that of the A_4_C_6_EP coating. It is indicated that the addition of graphite nanosheets further enhances the corrosion resistance of the coating.

**Figure 5:**
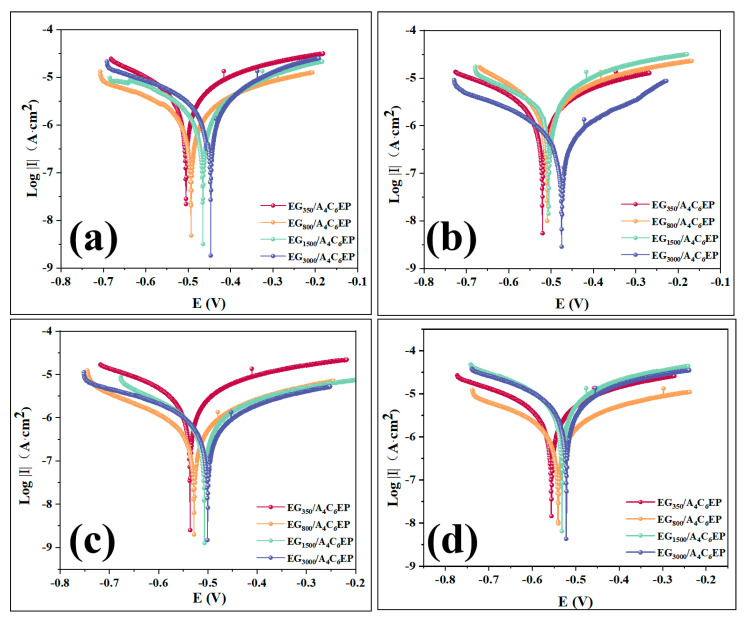
Tafel images of EG_350–3000_A_4_C_6_EP coatings at 7/14/30/60 days of immersion (**a**–**d**).

To examine polarization reactions occurring on the surfaces of four coatings and validate the impedance and Bode principles, Tafel polarization tests were performed on these coatings after immersion in the NaCl solution for various durations [24]. The results are shown in Figure 5. By analyzing the intersection of the cathodic and anodic polarization curves on the Tafel plot, corrosion potential (Ecorr) and corrosion current density (Icorr) can be determined, with the specific values provided in Table S2. For coatings immersed for the same duration, those with higher Ecorr and lower Icorr values exhibit better corrosion resistance [32].

After 7 days of immersion in NaCl solution (Figure 5a), the EG_3000_/A_4_C_6_EP coating exhibits a corrosion potential of −448.36 mV, a corrosion current density of 3.00 × 10^−7^ A·cm^−2^, and a corrosion rate of 96.81%. Compared to the other three coatings, the cathodic and anodic polarization curves of the EG_3000_/A_4_C_6_EP coating shift toward the lower right, indicating the superior inhibition of cathodic and anodic reactions. Additionally, as the potential changes, the electrode surface becomes more stable, demonstrating the best corrosion resistance among the coatings tested. After 14 days of immersion (Figure 5b), the corrosion potential of the EG_3000_/A_4_C_6_EP coating decreases to −499.37 mV, with a corrosion current density of 5.87 × 10^−7^ A·cm^−2^ and a corrosion rate of 94.50%. Although the corrosion potential decreases and corrosion current increases compared to the 7-day mark, changes are minimal, indicating the coating’s stable corrosion resistance over time. After 30 days of immersion (Figure 5c), the corrosion potential further decreases to −502.35 mV, while the corrosion current density slightly increases to 5.96 × 10^−7^ A·cm^−2^, resulting in a corrosion rate of 93.66%. Despite these changes, the EG_3000_/A_4_C_6_EP coating still exhibits a higher corrosion potential and lower corrosion current density than the other coatings, further demonstrating its superior corrosion resistance. After 60 days of immersion (Figure 5d), the EG_3000_/A_4_C_6_EP coating maintains the highest corrosion potential and lowest corrosion current density, with a corrosion rate of 86.38%, reflecting only a 10% decrease from the 7-day value. This sustained performance highlights the coating’s exceptional long-term stability and corrosion protection, consistent with the findings from impedance and Bode analyses.

#### 3.2.2. Salt Spray Test

**Figure 6:**
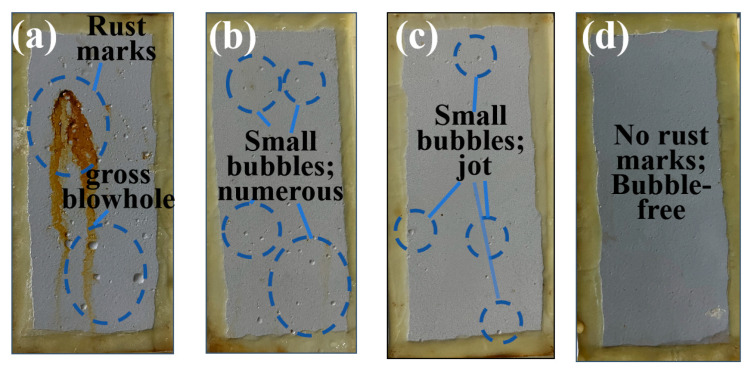
Salt spray test at 2880 h and images of EG_350_A_4_C_6_EP coating (**a**), EG_800_A_4_C_6_EP coating (**b**), EG_1500_A_4_C_6_EP coating (**c**), and EG_3000_A_4_C_6_EP coating (**d**).

To visually assess the corrosion resistance of the coatings and validate the results of the electrochemical tests, the four coatings were uniformly applied to treated Q235 steel using a coater. The samples were then placed in a NaCl salt spray test chamber under constant temperature and pressure for testing. The results after 2880 h are shown in Figure 6. The corrosion resistance of coatings was evaluated in accordance with the ISO 4628-2 and ISO 4628-3 standards, utilizing rust coverage area and blister density as key metrics for corrosion rating classification. Severe corrosion is demonstrated by substantial rust formation combined with high-density, large-volume blistering. Conversely, minimal blistering and the absence of rust indicate mild corrosion. A complete lack of both blisters and rust spots confirms the coating remains uncorroded under tested conditions.

After 2880 h in the salt spray chamber, the EG_350_A_4_C_6_EP coating exhibits small rust spots and numerous large bubbles on its surface (Figure 6a), indicating a decline in corrosion resistance after prolonged exposure. Meanwhile, the EG_800_A_4_C_6_EP coating (Figure 6b) shows no yellowish-brown rust marks, though a substantial number of bubbles appear, suggesting an improvement in corrosion resistance compared to the EG_350_A_4_C_6_EP coating. Compared to the EG_800_A_4_C_6_EP coating, the EG_1500_A_4_C_6_EP coating does not form large bubbles after salt spray corrosion (Figure 6c), but mild blistering is observed on it, indicating a further improvement in corrosion resistance. After the salt spray test, the surface of the EG_3000_A_4_C_6_EP coating shows no rust marks or bubbles (Figure 6d), demonstrating that it exhibits the best corrosion resistance among the four coatings, which is consistent with the electrochemical results.

**Figure 7:**
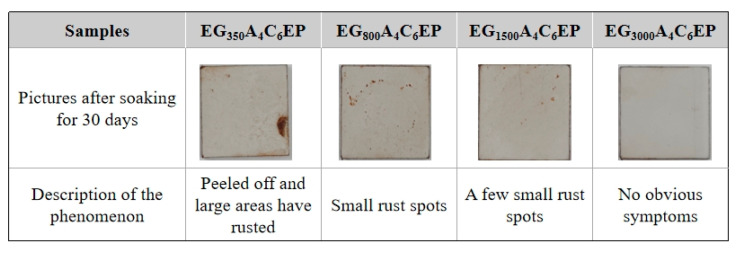
Images of the four coatings after 30 days of immersion.

To assess the durability of the four coatings, they were applied to small Q235 steel plates, cured, and then immersed in a 3.5 wt% NaCl solution for 30 days, and the results are shown in Figure 7. The EG_350_A_4_C_6_EP coating exhibits peeling and large rust stains after 30 days of immersion. Meanwhile, the EG_800_A_4_C_6_EP coating shows numerous small rust spots, while the EG_1500_A_4_C_6_EP coating displays considerably fewer rust spots. The EG_3000_A_4_C_6_EP coating demonstrates no peeling or rust spots, indicating its superior durability.

### 3.3. Mechanical Wear Test

**Figure 8:**
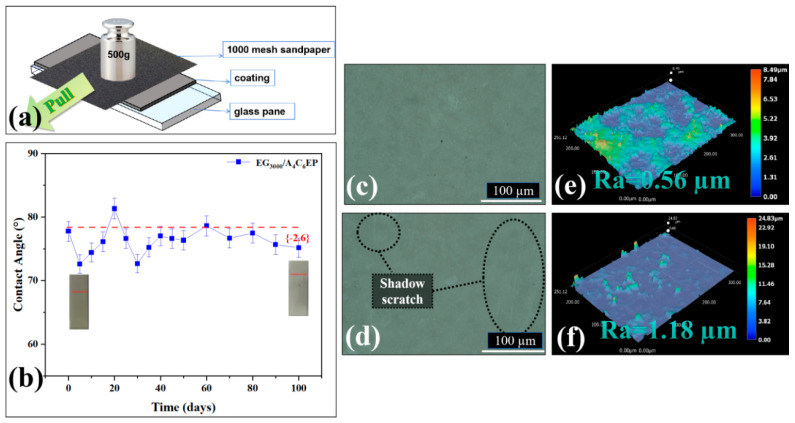
Mechanical wear test diagram (**a**). Contact angle variation diagram (**b**). Schematic diagram of surface before friction (**c**). Diagram of surface after friction (**d**). Surface roughness before friction (**e**). Surface roughness after friction (**f**).

To evaluate the mechanical abrasion resistance of the coating, an abrasion test was performed by applying pressure with a sandpaper. Changes in the contact angle of the coating were monitored, and the coating surface condition was examined using a microscope and a roughness tester to assess its mechanical abrasion resistance. The results are presented in Figure 8.

During the first five friction cycles, the contact angle of the coating decreases considerably. This is attributed to frictional damage caused under the application of extreme pressure and relatively rough sandpaper, along with some measurement errors, substantially decreasing the coating’s hydrophobic angle. Between the fifth and twentieth cycles, the hydrophobic angle of the coating gradually increases, even surpassing its original value. This occurs because, as the friction cycles progress, errors are minimized, and the coating surface does not experience considerable damage during the early cycles. As the friction cycles progress, the roughness of the coating increases considerably, increasing its hydrophobic angle. Although the hydrophobic angle of the coating fluctuates with an increasing number of cycles, it generally exhibits a decreasing trend in variability. This can be attributed to the surface damage caused during the abrasion cycles, gradually decreasing the hydrophobic angle over time. After 100 cycles, the hydrophobic angle of the coating decreases by only 2.6° (Figure 8b), and the wear rate is merely 6.53% (Table S4), demonstrating its excellent mechanical abrasion resistance. A comparison of the surface condition of the coating before and after the friction tests (Figure 8c,d) reveals, under microscopic observation, that after 100 cycles, the coating surface exhibits only minor scratches with no considerable damage. Roughness increases by only 0.62 µm (Figure 8e,f). This confirms that although mechanical abrasion causes some damage, it is minimal, further supporting the coating’s excellent mechanical abrasion resistance.

### 3.4. Coating Corrosion Resistance Mechanism Test

**Figure 9:**
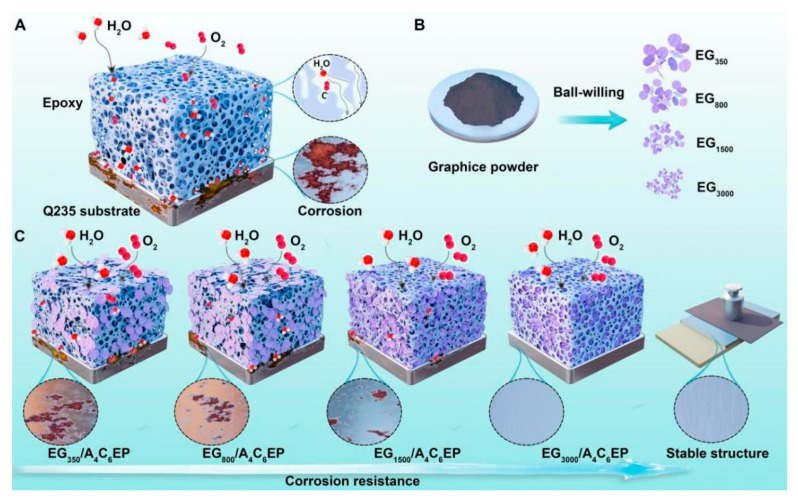
Diagram of corrosion resistance mechanism of coating.

To comprehensively study the corrosion resistance of the coatings, simulations were performed to explore their corrosion resistance mechanisms, and the results are shown in Figure 9. The pure EP resin coating, owing to its inherent micropores and cracks (Figure 9A), is susceptible to infiltration by corrosive particles, such as oxygen and water molecules, from air through these defects, leading to the contact of these particles with the substrate and the initiation of corrosion. Graphite powder, subjected to ball milling and varying centrifugation rates, is mechanically exfoliated into graphite nanosheets of different sizes (Figure 9B). When graphite nanosheets are incorporated into the EP coating, they fill micropores and defects in the pure EP resin, improving its overall structure and blocking the pathways used by corrosive particles to reach the substrate. This enhances the coating’s corrosion resistance. Additionally, as graphite nanosheets are distributed in the EP coating, smaller nanosheets are more likely to penetrate the coating’s inherent defects, further improving its integrity and adhesion to nanosheets. This results in the superior stability and mechanical wear resistance of the coating.

When incorporating graphite nanosheets of different sizes into the EP resin coating (Figure 9C), large nanosheets tend to accumulate in the coating without filling its defects. This increases the likelihood of corrosive particles from air penetrating the coating and reaching the substrate, thereby leading to severe corrosion. Meanwhile, small graphite nanosheets can infiltrate the EP coating’s defects, adhering to microcracks and pores owing to the adhesive properties of the EP resin. This process helps repair the coating’s defects. This process effectively blocks the pathways used by corrosive media to reach the substrate, delaying corrosion reactions and thereby imparting excellent corrosion resistance to the coating. However, achieving optimal performance requires an appropriate concentration of graphene nanosheets. If the concentration is too low, nanosheets fail to adequately fill defects in the EP coating. Meanwhile, if the concentration is too high, nanosheets aggregate, increasing their size and reducing their effectiveness. Therefore, only the optimized concentration of nanosheets can provide the best barrier effect, ensuring the superior protective performance of the coating.

## 4. Conclusions

This study verifies the size effect of graphite nanosheets in hydrophilic coatings and develops a corrosion inhibitor delivery system based on a hydrophilic EP coating package. These nanosheets, produced through cost-effective mechanical stripping via ball milling and gradient centrifugation, vary in size. EIS and Tafel polarization analyses confirm that the EG_3000_A_4_C_6_EP coating, incorporating optimally sized EG_3000_ nanosheets (1.01 ± 0.1 μm), provides effective corrosion inhibition. This coating forms a nanonetwork that fills microcracks in the mullite/kaolin particles, thereby hindering the diffusion of corrosive media. Additionally, salt spray testing and corrosion product analysis further demonstrate that smaller graphene nanosheets more effectively block corrosion particles. EG_3000_ also contributes to forming a stable and robust structure between microkaolin/mullite and the EP coating, enhancing the coating’s mechanical wear resistance. The use of easily controllable graphene nanosheets demonstrates that their corrosion inhibition effect remains consistent regardless of changes in coating hydrophobicity. The exceptional properties of graphene nanosheets offer a straightforward and promising approach to anticorrosion engineering, with broad application potential.

## Data Availability

The authors declare that they have no known competing financial interests or personal relationships that could have appeared to influence the work reported in this paper.

## Supplementary Materials

The following supporting information can be downloaded at https://www.mdpi.com/article/10.3390/polym17131803/s1, Figure S1: SEM image of A_4_C_6_EP; Figure S2: SEM and Contact Angle image of EG_350_A_4_C_6_EP (a), EG_800_A_4_C_6_EP (b), EG_1500_A_4_C_6_EP (c), EG_3000_A_4_C_6_EP (d); Figure S3: XPS image of EG_3000_A_4_C_6_EP; Figure S4: XPS image of Kaolin; Figure S5: XPS image of Mullite; Figure S6: XPS image of EG_3000_; Figure S7: XPS image of Epoxy; Figure S8: Equivalent electric circuits of the collected EIS results; Figure S9: EIS/Bode/Tafel image of EG_350_A_4_C_6_EP/A_4_C_6_EP coatings at 7 days of immersion (a), (b) and (c); Table S1: EIS data of EG_350~3000_A_4_C_6_EP coatings at 7/14/30/60 days of immersion; Table S2: Tafel data of EG_350~3000_A_4_C_6_EP coatings at 7/14/30/60 days of immersion; Table S3: Anticorrosion properties of mullite/kaolin reinforced WEP; Table S4: The thickness and wear rate data of the coating
