# Supplementary material for: Multifunctional Graphite Nanosheet–Hydrophilic Epoxy Anticorrosion Coatings via Size Confinement of Exfoliated Graphite
Source: Polymers (Basel). 2025 Jun 28;17(13):1803. doi: 10.3390/polym17131803 (PMC12252152; doi:10.3390/polym17131803)
Supplement: Supplementary file 1 [file polymers-17-01803-s001.zip › polymers-3701614-supplementary.pdf]

Supporting information

Huachao Ma 1,2, Xuyang Zhang 2,3, Dongxing Zhang 2, Yizhan Peng 1, Detian Wan 2, Tai Peng 1,* and Kuilin Lv 2,*
1 College of Materials Science and Engineering, Jiamusi University, Jiamusi 154007, China; m18769082751@163.com (H.M.); 18753019632@163.com (Y.P.)
2 China Testing & Certification International Group Co., Ltd., Room, Beijing 100024, China; zhang-dongxing@ctc.com (D.Z.); dtwan@ctc.ac.cn (D.W.)
3 School of Civil and Transportation Engineering, Beijing University of Civil engineering and Architecture, Beijing 102616, China; 18736768339@163.com (X.Z.)
* Correspondence: pt@jmsu.edu.cn (T.P.); lv_k_l@163.com (K.L.)

2. Materials and Methods

2.1 Materials

Kaolin (CK, 5 μm), mullite (AM, > 200 items), poly(vinyl butyral) (PVB, M.W.90000-120000), graphite powder (EG, 99.9%), epoxy resin (EP, E-44), polyamide curing agent (PA, 650), N-vinylpyrrolidone (NVP, 98%) were obtained from Shanghai Maclin Biochemical Technology Co., Ltd. Ethanol was obtained from Modern Oriental Technology Development Co., Ltd.

2.2 Synthesis of the Different Sizes of Graphite Sheets

Different sizes of graphite nanosheets were prepared by wet ball milling and gradient centrifugation. EG (4 g), 2 and 0.2 mm zirconium dioxide microspheres (1:1) and NVP (100 mL) were added to the 250mL ball mill tank for 175 h of continuous ball milling. After the reaction, the dispersion was collected and centrifuged for 10 min at a quarter-speed of 350 r min^{-1}. The supernatant was collected and stored as sample A (EG_{350}). A quarter of the dispersion was centrifuged for 10 min at 800r min^{-1}, and the resulting supernatant was labeled as sample B (EG_{800}) and stored. Similarly, a quarter of the dispersed solution is centrifuged for 10 min at 1500 r min^{-1}; The obtained supernatant was labeled as sample C (EG_{1500}). The remaining dispersion was centrifuged for 10 min at 3000 r min^{-1}. The resulting supernatant was labeled as sample D (EG_{3000}). Its concentration is about ≤0.065wt%.

2.3 Preparation of composite coatings

Before applying the superhydrophobic film, the steel plate (20 mm W x 20 mm L x 1 mm T) is polished with 150 mesh sandpaper to remove any oil and rust. Ultrasonic cleaning was carried out with distilled water and anhydrous ethanol

respectively, and the steel plate was cleaned for 10 min, and then dried in the oven.

2 mL A (B, C, D) solution, 2 mL xylene, 18 mL anhydrous ethanol, 4 g mullite, 6 g kaolin, 1 g PVB and 0.4 g castor oil were poured into a 250 mL ball mill tank containing several 0.2mm and 2 mm ZrO_2 pellets (weight ratio 1:1). After continuous ball milling for 4 h, remove the slurry. Weigh 10 g of slurry, add 1 g of epoxy resin and pour it into the beaker, after 15 min of ultrasound, stir it into the stirring table for 30 min, weigh 1 g of polyamide resin and pour it into the beaker, after 15 min of ultrasound, stir it into the stirring table for 30 min, absorb 100 μL of anti-flash rust agent with a pipette and pour it into the beaker. After stirring for 5 min, EG_x/A_4C_6EP coated slurry was obtained, which was coated with 150 mm wire rod. After coating, it was left to dry at room temperature for 24 h, until completely dried, and then the second coating was performed. After drying at room temperature for 24 h, the rosin and paraffin (weight ratio: 1: 1) Mix heating, seal the coated plate, and dry at room temperature for 12 h to obtain EG_{350}/A_4C_6EP board, EG_{800}/A_4C_6EP board, EG_{1500}/A_4C_6EP board and EG_{3000}/A_4C_6EP board.

2.4 Electrochemical measurements

Corrosion resistance is tested by electrochemistry and salt spray. Electrochemical experiments were conducted on the DH7000 electrochemical workstation. The reference, and auxiliary electrodes were coated with a saturated calomel electrode and platinum electrode. The effective area of the working electrode was 6.16 cm^2, and the corrosion potential was measured with a step size of 2 s. Long-term electrochemical impedance spectroscopy (EIS) was used to analyze the disturbances at an amplitude of 10 mV and a frequency range of 100 mHz–100 kHz. Before measurements, the coating was immersed in a 3.5 wt% NaCl solution until a stable open circuit potential was established. Tafel potential polarization tests were performed at a constant current rate of 1 mv/s for calculating the corrosion potential (E_{corr}) and corrosion current through Tafel extrapolation of density (I_{corr}). The EIS results were analyzed using the Thales software.

2.5 Salt spray corrosion testing (SSCT)

Samples for salt spray corrosion testing (SSCT) were exposed to a 3.5 wt% NaCl solution in a cabinet placed on an iron rack following the guidelines of ASTM B117[50]. The pressure of the atomized normal saline was maintained at 70–120 kPa, and the temperature inside the cabinet was controlled at 35℃ ± 2℃. The samples were then categorized based on their recorded SSCT times (7, 14, 21, 28, and 30 days), dried using warm ambient air, and promptly sealed.

2.6 Evaluation of mechanical durability

The abrasion resistance test was verified by sandpaper friction test. The friction resistance is reflected in the number of webs of sandpaper and the weight of the weight covered on the sandpaper. In the sandpaper test, the superhydrophobic film coated glass substrate is contacted with 1000 well sandpaper. The sandpaper and the substrate are applied together to a 500 gram load, and an external force pushes it horizontally along the scale to 3 cm, forming a cycle. It was measured every 5 wear cycles for the first 50 cycles and every 10 wear cycles thereafter. After 100 cycles, the surface Angle and hydrophobic Angle of the hydrophilic membrane were observed.

3. Results and discussion

3.1 Structure and morphology

3.1.1 Morphology characterization of samples and coatings

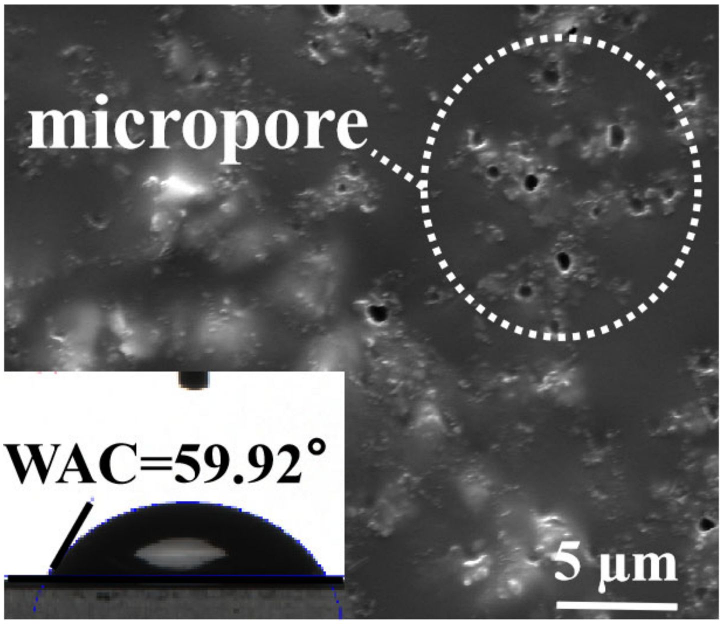

Fig. S1 SEM image of A_4C_6EP

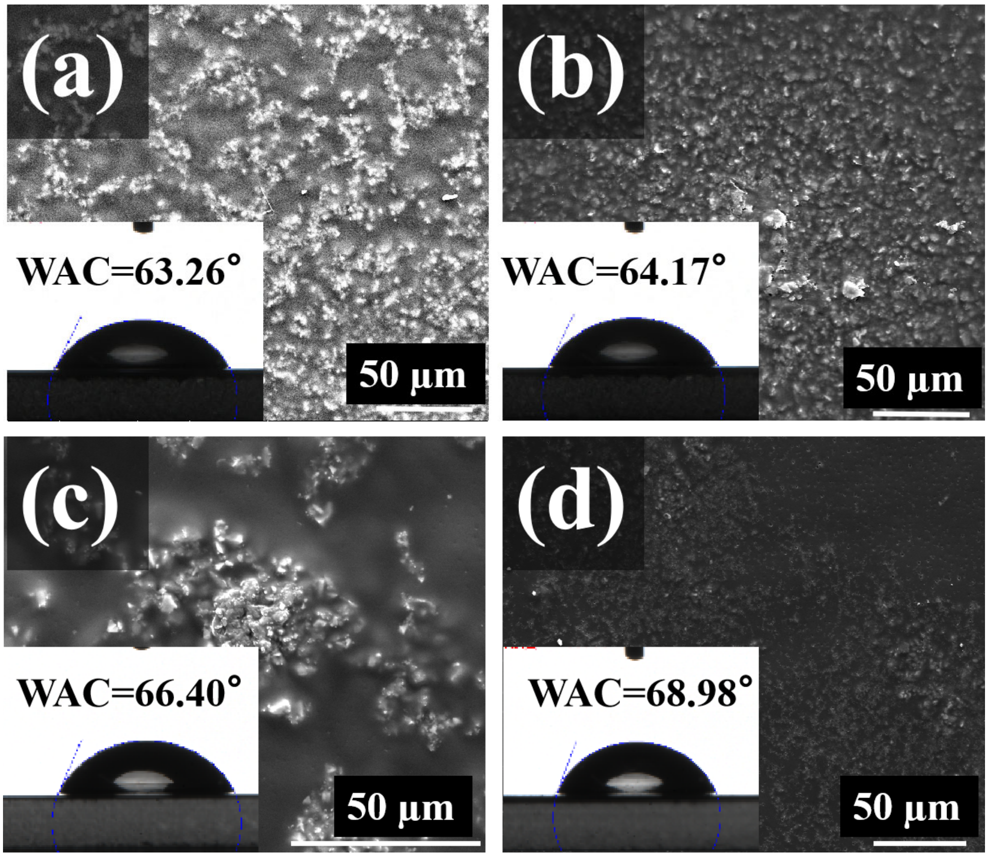

Fig. S2 SEM and Contact Angle image of $EG_{350}A_4C_6EP$ (a), $EG_{800}A_4C_6EP$ (b), $EG_{1500}A_4C_6EP$ (c), $EG_{3000}A_4C_6EP$ (d).

3.1.2. Structural characterization of samples and coatings

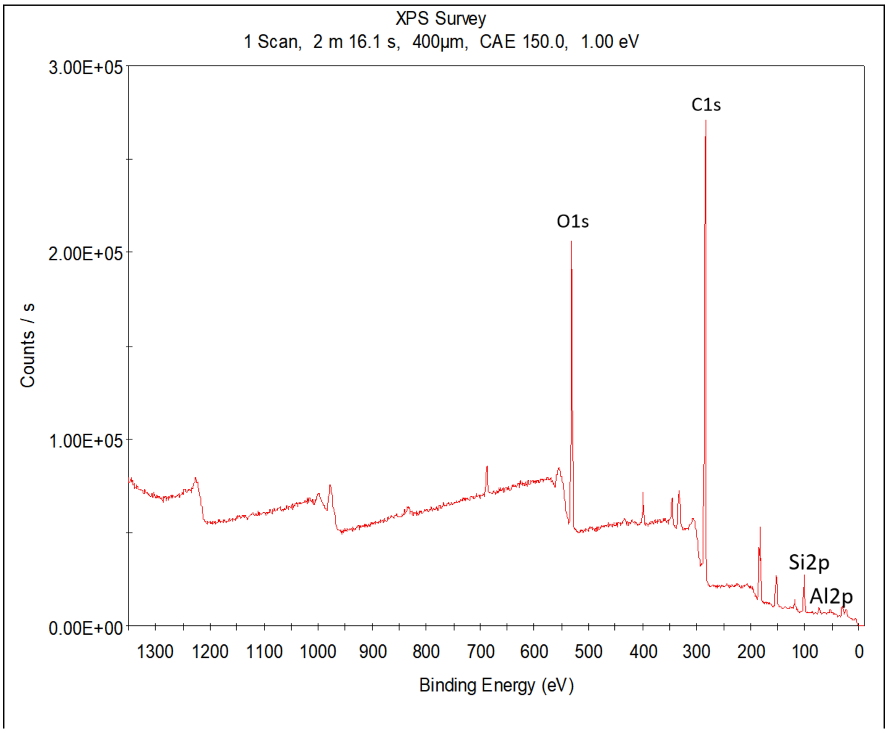

Fig. S3 XPS image of $EG_{3000}A_4C_6EP$

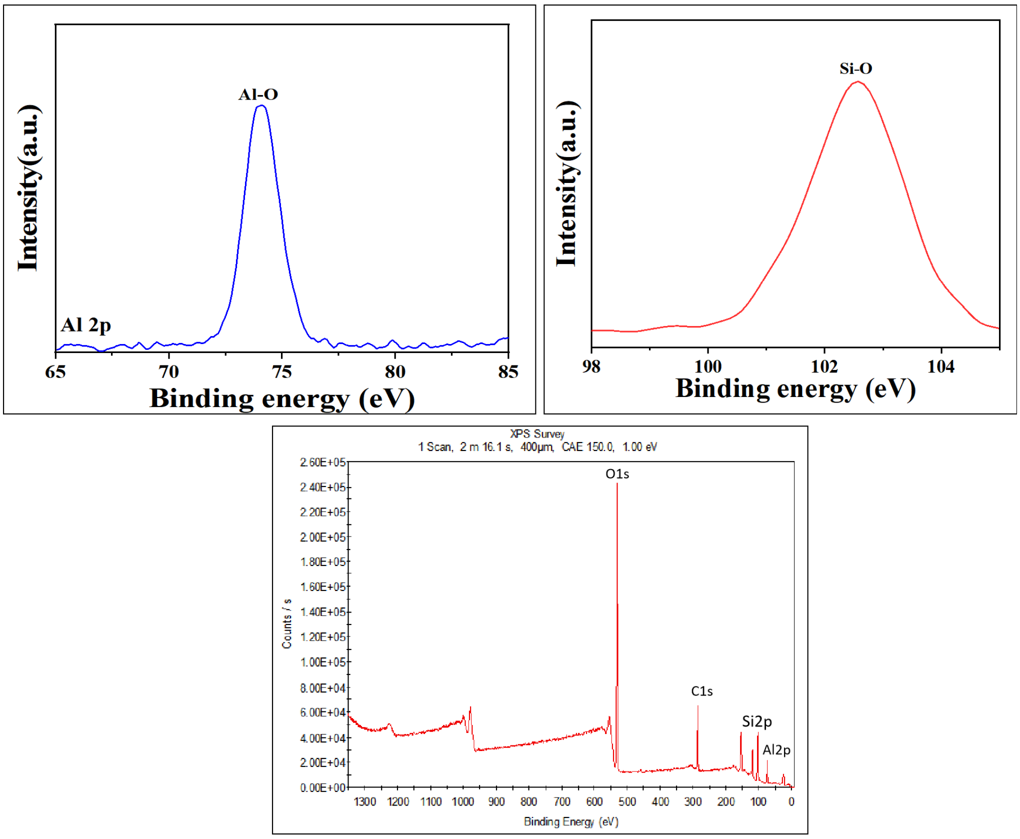

Fig. S4 XPS image of Kaolin

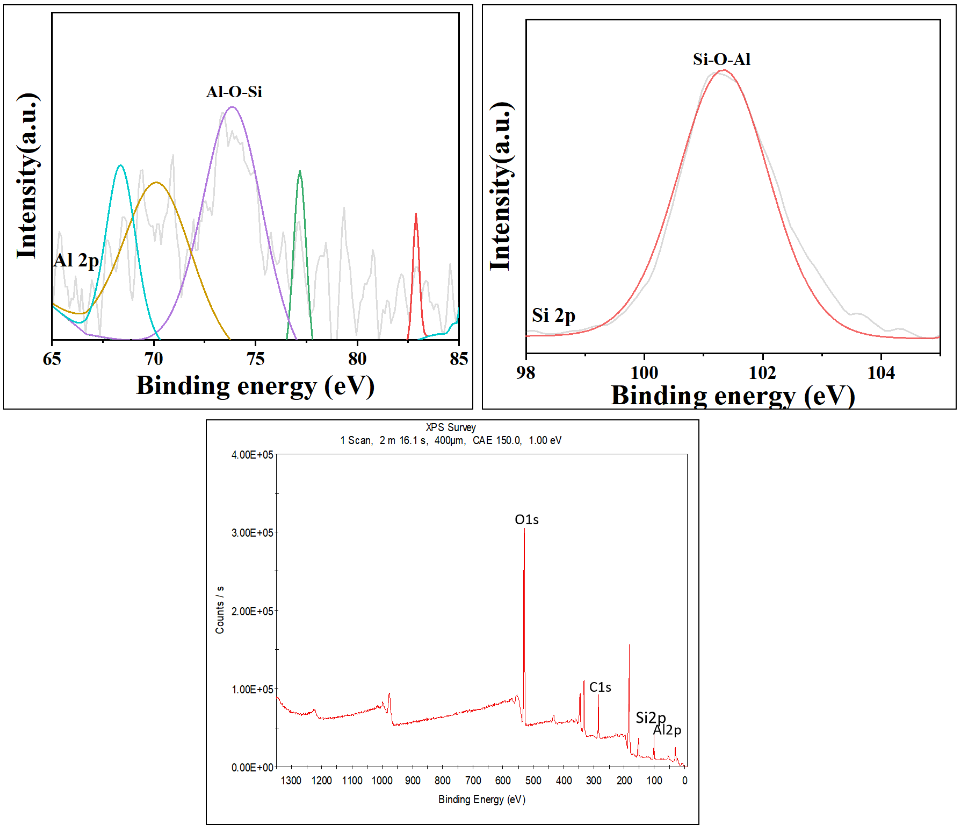

Fig. S5 XPS image of Mullite

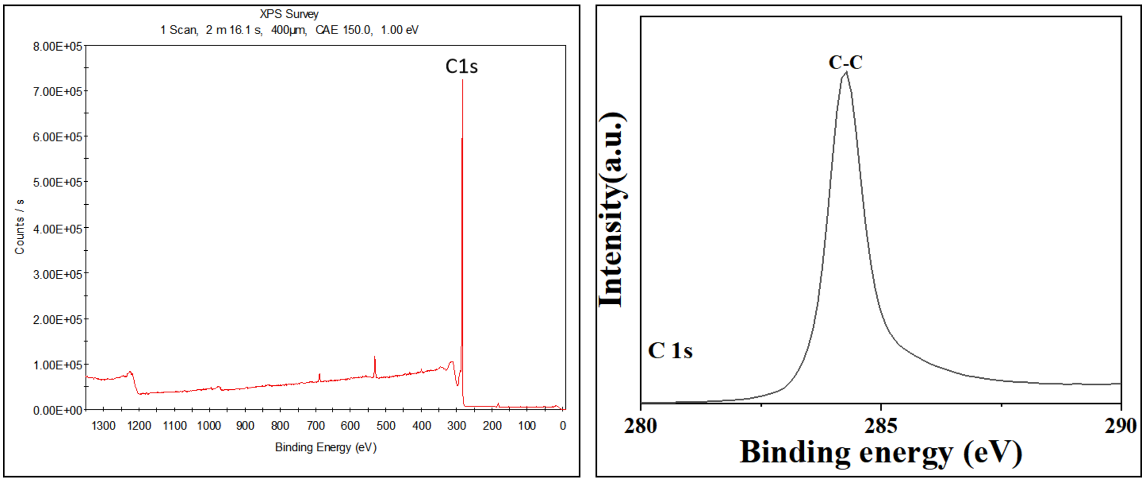

Fig. S6 XPS image of EG_{3000}

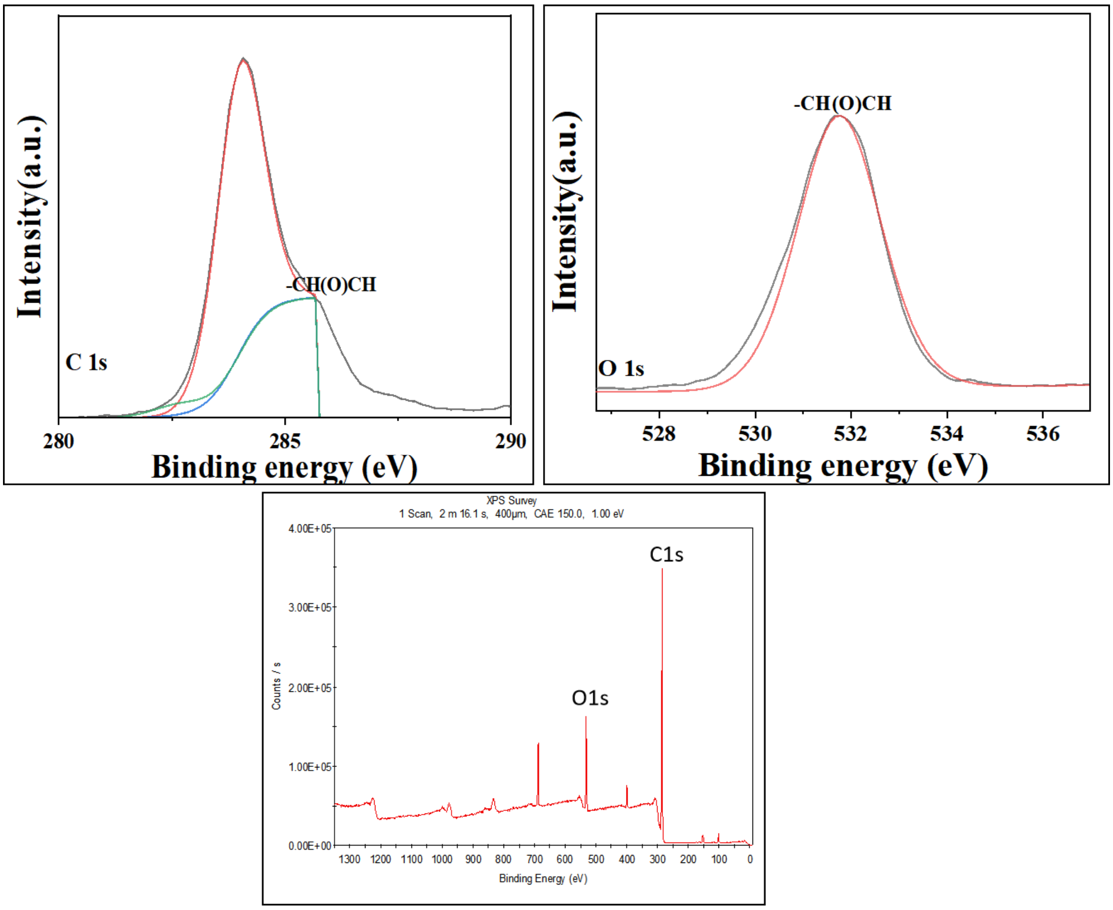

Fig. S7 XPS image of Epoxy

3.2. Corrosion resistance test

3.2.1. Electrochemical measurement

Table S1 EIS data of $EG_{350\sim3000}A_4C_6EP$ coatings at 7/14/30/60 days of immersion

Time (day)	Samples	Rs (Ω•cm^2)	C (Ω^{-1}•cm^{-2}•sn)	Rp (Ω•cm^2)
7	A_4C_6EP	7.99×10^4	5.96×10^{-11}	4.69×10^7
	EG_{350}/A_4C_6EP	1.03×10^5	1.20×10^{-10}	6.81×10^7
	EG_{800}/A_4C_6EP	1.01×10^5	1.15×10^{-10}	7.96×10^7
	EG_{1500}/A_4C_6EP	1.32×10^5	7.12×10^{-11}	8.45×10^7

	EG$_{3000}$/A$_4$C$_6$EP	1.12×10^{5}	1.14×10^{-10}	9.18×10^{7}
	EG$_{350}$/A$_4$C$_6$EP	0.62×10^{5}	8.16×10^{-11}	6.05×10^{7}
14	EG$_{800}$/A$_4$C$_6$EP	7.91×10^{5}	2.34×10^{-10}	6.78×10^{7}
	EG$_{1500}$/A$_4$C$_6$EP	0.72×10^{5}	1.67×10^{-10}	7.96×10^{7}
	EG$_{3000}$/A$_4$C$_6$EP	1.69×10^{5}	1.07×10^{-10}	8.95×10^{7}
	EG$_{350}$/A$_4$C$_6$EP	0.86×10^{5}	1.51×10^{-10}	5.09×10^{7}
30	EG$_{800}$/A$_4$C$_6$EP	1.77×10^{5}	1.53×10^{-10}	6.29×10^{7}
	EG$_{1500}$/A$_4$C$_6$EP	0.63×10^{5}	8.71×10^{-11}	6.51×10^{7}
	EG$_{3000}$/A$_4$C$_6$EP	7.39×10^{5}	2.17×10^{-10}	8.51×10^{7}
	EG$_{350}$/A$_4$C$_6$EP	1.42×10^{5}	8.61×10^{-11}	3.00×10^{7}
60	EG$_{800}$/A$_4$C$_6$EP	0.95×10^{5}	8.61×10^{-11}	3.94×10^{7}
	EG$_{1500}$/A$_4$C$_6$EP	0.64×10^{5}	8.18×10^{-11}	4.97×10^{7}
	EG$_{3000}$/A$_4$C$_6$EP	1.01×10^{5}	1.24×10^{-10}	5.82×10^{7}

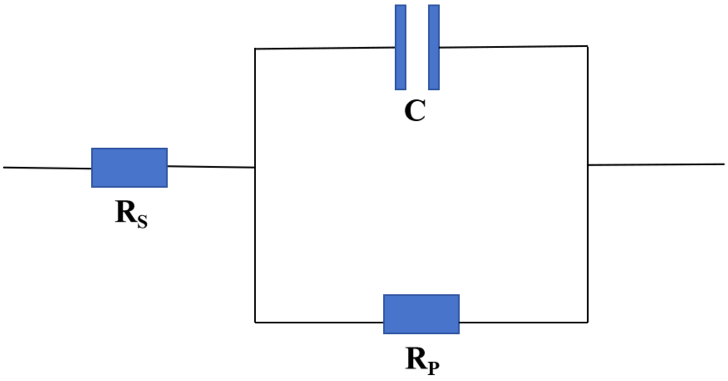

Fig. S8 Equivalent electric circuits of the collected EIS results.

Table S2 Tafel data of EG$_{350\sim3000}$A$_4$C$_6$EP coatings at 7/14/30/60 days of immersion

Time (day)	Samples	Ecorr/mV	Icorr/A•cm^2	η/%
	Q235	-631.36	9.40×10^{-6}	——
7	A$_4$C$_6$EP	-528.87	2.79×10^{-6}	70.31
	EG$_{350}$/A$_4$C$_6$EP	-507.88	9.19×10^{-7}	90.22

	EG_{800}/A_4C_6EP	-490.90	3.54×10^{-7}	96.23
	EG_{1500}/A_4C_6EP	-463.69	3.08×10^{-7}	96.72
	EG_{3000}/A_4C_6EP	-448.36	3.00×10^{-7}	96.81
	EG_{350}/A_4C_6EP	-521.44	1.19×10^{-6}	87.34
14	EG_{800}/A_4C_6EP	-511.97	1.16×10^{-6}	87.66
	EG_{1500}/A_4C_6EP	-537.96	1.92×10^{-6}	79.57
	EG_{3000}/A_4C_6EP	-499.37	5.87×10^{-7}	94.50
	EG_{350}/A_4C_6EP	-537.96	2.01×10^{-7}	78.62
30	EG_{800}/A_4C_6EP	-527.22	1.47×10^{-6}	84.36
	EG_{1500}/A_4C_6EP	-509.15	1.16×10^{-6}	87.66
	EG_{3000}/A_4C_6EP	-502.35	5.96×10^{-7}	93.66
	EG_{350}/A_4C_6EP	-553.13	3.71×10^{-6}	60.53
60	EG_{800}/A_4C_6EP	-540.54	2.02×10^{-6}	78.51
	EG_{1500}/A_4C_6EP	-531.69	1.56×10^{-6}	83.40
	EG_{3000}/A_4C_6EP	-522.26	1.28×10^{-6}	86.38

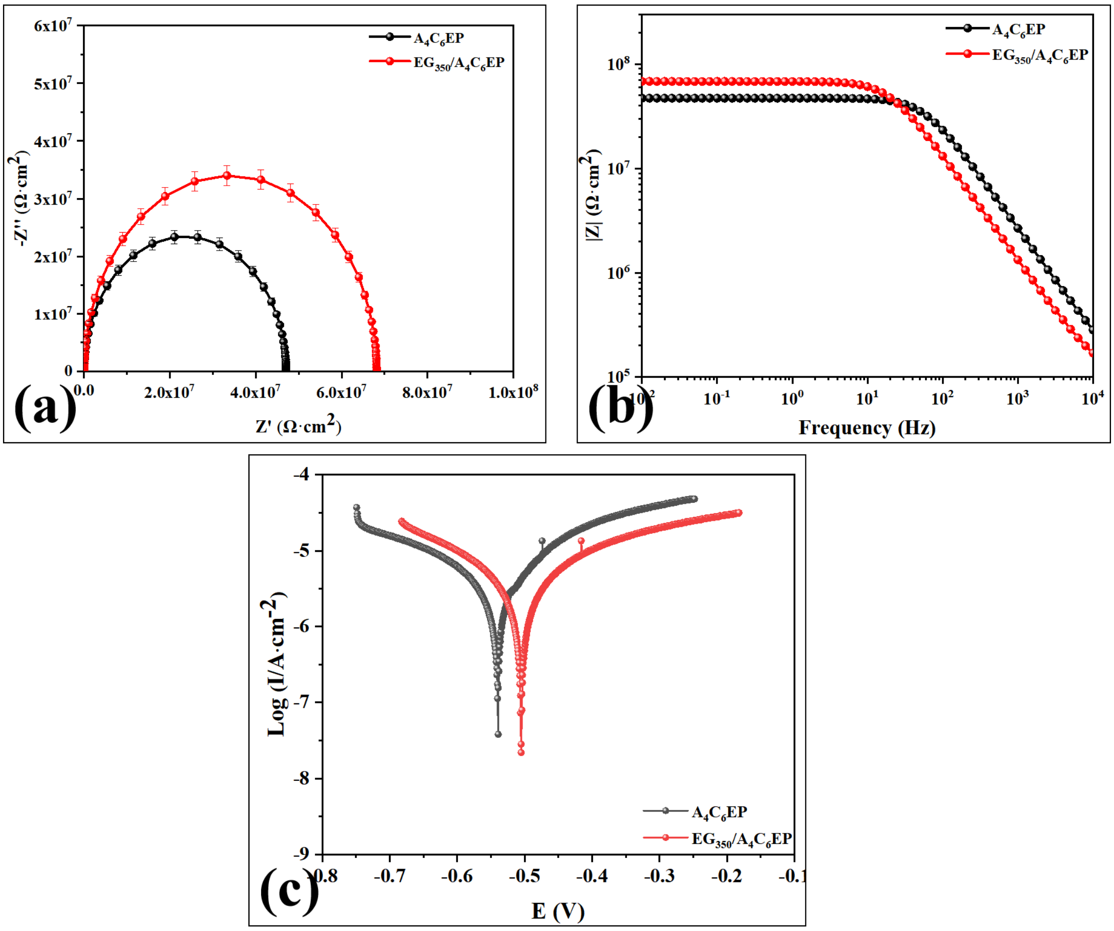

Fig. S9 EIS/Bode/Tafel image of $EG_{350}A_4C_6EP/A_4C_6EP$ coatings at 7 days of immersion (a), (b) and (c)

Table S3. Anticorrosion properties of mullite/kaolin reinforced WEP.

Samples	Immersion duration in 3.5% NaCl (days)	Coating thickness (μm)	Rc (Ω cm²)	CPEc (F)	Reference
$EG_{1500}SO_{0.1}SN_{0.9}EP$	30 (min)	25 ± 0.5	4.65×10^{6}	7.54×10^{-8}	2
$AO_{0.3}AN_{0.7}EP$	7	25 ± 1	2.42×10^{7}	7.22×10^{-11}	3
$EG_{3000}A_4C_6EP$	7	25 ± 2	9.18×10^{7}	1.14×10^{-10}	This Work

3.3. Mechanical wear test

Table S4. The thickness and wear rate data of the coating

Material / Test	EG_{350}/A_4C_6EP	EG_{800}/A_4C_6EP	EG_{1500}/A_4C_6EP	EG_{3000}/A_4C_6EP
Coating thickness (μm)	62.12	60.24	61.83	61.23
wear rate(%)	15.23	13.12	10.28	6.53
